# Supplementary material for: Polyketide synthase gene clusters in the ericoid mycorrhizal fungus Oidiodendron maius: genome-wide prediction, expression profiling, and functional characterization of knockout mutants
Source: Front Plant Sci. 2026 Sep 16;17:1924430. doi: 10.3389/fpls.2026.1924430 (PMC13623903; doi:10.3389/fpls.2026.1924430)
Supplement: Supplementary file 1 [file SupplementaryFile1.zip › Supplementary Materials/Supplementary Folder 1.zip!/Oidma1_AssemblyScaffolds_Repeatmasked/knownclusterblast/cluster19/jgi.p_Oidma1_28493_mibig_hits.html]

| MIBiG Protein | Description | MIBiG Cluster | MiBiG Product | % ID | % Coverage | BLAST Score | E-value |
| --- | --- | --- | --- | --- | --- | --- | --- |
| CRG85577.1 | hypothetical\_protein | BGC0001402 | Cyclochlorotine\_biosynthetic\_gene\_cluster | 30 | 93.1729518856 | 569 | 1.6e-161 |
| EPE34342.1 | P-loop\_containing\_nucleoside\_triphosphate\_hydrolase | BGC0001035 | Pneumocandin\_biosynthetic\_gene\_cluster | 30 | 61.7685305592 | 351 | 6.8e-96 |
| AMM63163.1 | AniL | BGC0001371 | Echinocandin\_B\_biosynthetic\_gene\_cluster | 27 | 59.8829648895 | 346 | 2.2e-94 |
| AFT91383.1 | EcdL | BGC0000338 | Echinocandin\_B\_biosynthetic\_gene\_cluster | 27 | 60.0130039012 | 329 | 2.8e-89 |
| OAQ83754.1 | ABC\_multidrug\_transporter | BGC0001358 | Leucinostatins\_biosynthetic\_gene\_cluster | 28 | 63.9141742523 | 294 | 7.5e-79 |
| AAN74822.1 | Fum19p | BGC0000062 | Fumonisin\_biosynthetic\_gene\_cluster | 25 | 92.5877763329 | 280 | 1.5e-74 |
| ACB12564.1 | Fum19 | BGC0000063 | Fumonisin\_biosynthetic\_gene\_cluster | 27 | 63.6540962289 | 268 | 5.8e-71 |
| ACQ63630.1 | ABC\_multidrug\_resistance\_transporter | BGC0000875 | Caprazamycin\_biosynthetic\_gene\_cluster | 26 | 60.9232769831 | 231 | 6e-60 |
| AMR44284.1 | ABC\_transporter | BGC0001398 | Phomopsins\_biosynthetic\_gene\_cluster | 25 | 65.2795838752 | 223 | 1.3e-57 |
| ADC96668.1 | ABC\_transporter | BGC0001076 | Liposidomycin\_biosynthetic\_gene\_cluster | 24 | 67.6853055917 | 188 | 5.8e-47 |
| CAC20925.1 | PimB\_protein | BGC0000125 | Pimaricin\_biosynthetic\_gene\_cluster | 29 | 32.7698309493 | 174 | 1.1e-42 |
| ADX66465.1 | ScnB | BGC0000108 | Natamycin\_biosynthetic\_gene\_cluster | 29 | 32.6397919376 | 173 | 1.9e-42 |
| AAF71763.1 | NysG | BGC0000115 | Nystatin\_biosynthetic\_gene\_cluster | 29 | 31.9245773732 | 164 | 9e-40 |
| CAB38879.1 | putative\_ABC\_transporter | BGC0000315 | Calcium-dependent\_antibiotic\_biosynthetic\_gene\_cluster | 27 | 36.8660598179 | 158 | 8.4e-38 |
| AAK73498.1 | AmphG | BGC0000015 | Amphotericin\_biosynthetic\_gene\_cluster | 26 | 32.1196358908 | 153 | 2.1e-36 |
| ABV83218.1 | CppG | BGC0000116 | Nystatin-like\_Pseudonocardia\_polyene\_biosynthetic\_gene\_cluster | 27 | 32.5097529259 | 151 | 1e-35 |
| CAF31837.1 | putative\_hygromycin\_B\_exporter | BGC0000699 | Hygromycin\_B\_biosynthetic\_gene\_cluster | 26 | 34.9154746424 | 150 | 1.8e-35 |
| CAC20924.1 | PimA\_protein | BGC0000125 | Pimaricin\_biosynthetic\_gene\_cluster | 31 | 25.8127438231 | 149 | 3.9e-35 |
| CAG38700.1 | putative\_ABC-transporter,\_ATP-binding\_protein | BGC0000708 | Lividomycin\_biosynthetic\_gene\_cluster | 28 | 37.1261378414 | 149 | 3.9e-35 |
| ADX66464.1 | ScnA | BGC0000108 | Natamycin\_biosynthetic\_gene\_cluster | 30 | 25.6827048114 | 147 | 1.5e-34 |
| ANP43732.1 | ThsT | BGC0001391 | Thusin\_biosynthetic\_gene\_cluster | 25 | 39.9869960988 | 143 | 2.1e-33 |
| CAG34715.1 | putative\_ABC\_transporter | BGC0000713 | Ribostamycin\_biosynthetic\_gene\_cluster | 26 | 32.5097529259 | 143 | 2.1e-33 |
| AIS24845.1 | Dst21 | BGC0001147 | Distamycin\_biosynthetic\_gene\_cluster | 28 | 32.6397919376 | 142 | 4.8e-33 |
| ALV82355.1 | ABC\_transporter | BGC0001370 | Lipopeptide\_8D1-1\_/\_lipopeptide\_8D1-2\_biosynthetic\_gene\_cluster | 26 | 36.9960988296 | 142 | 4.8e-33 |
| BAE07080.1 | possible\_ABC\_transporter | BGC0000693 | Butirosin\_biosynthetic\_gene\_cluster | 25 | 36.2808842653 | 141 | 6.2e-33 |
| CAI47637.1 | putative\_ABC-type\_aminoglycoside\_exporter | BGC0000692 | Apramycin\_biosynthetic\_gene\_cluster | 27 | 35.8257477243 | 141 | 1.1e-32 |
| BAI23327.1 | putative\_ABC-transporter | BGC0000949 | A-500359s\_biosynthetic\_gene\_cluster | 27 | 37.1261378414 | 140 | 1.8e-32 |
| AAX31531.1 | putative\_ABC\_transporter | BGC0000336 | Daptomycin\_biosynthetic\_gene\_cluster | 25 | 32.5097529259 | 139 | 3.1e-32 |
| BAJ05895.1 | putative\_ABC\_multidrug\_resistance\_transporter | BGC0000872 | A-90289\_biosynthetic\_gene\_cluster | 26 | 33.6801040312 | 138 | 5.3e-32 |
| BAH43768.1 | ABC\_transporter\_ATP-binding/permease\_protein | BGC0000452 | Tyrocidine\_biosynthetic\_gene\_cluster | 26 | 36.2808842653 | 137 | 1.2e-31 |
| AAF71764.1 | NysH | BGC0000115 | Nystatin\_biosynthetic\_gene\_cluster | 30 | 27.5682704811 | 136 | 3.4e-31 |
| ABV83219.1 | CppH | BGC0000116 | Nystatin-like\_Pseudonocardia\_polyene\_biosynthetic\_gene\_cluster | 29 | 24.9674902471 | 135 | 4.5e-31 |
| CAJ88629.1 | putative\_netropsin\_resistance\_protein\_subunit\_2 | BGC0000327 | Congocidine\_biosynthetic\_gene\_cluster | 26 | 32.8348504551 | 133 | 2.2e-30 |
| AHH99931.1 | ABC\_transporter\_protein | BGC0000002 | Aculeximycin\_biosynthetic\_gene\_cluster | 31 | 20.4811443433 | 133 | 2.2e-30 |
| ABW74893.1 | ABC\_transporter | BGC0000759 | Diutan\_polysaccharide\_biosynthetic\_gene\_cluster | 26 | 32.3797139142 | 133 | 2.9e-30 |
| ABD65953.1 | ABC\_transporter | BGC0000341 | Enduracidin\_biosynthetic\_gene\_cluster | 24 | 38.166449935 | 131 | 8.4e-30 |
| AAP57696.1 | putative\_secretion\_protein | BGC0000768 | Gellan\_polysaccharide\_biosynthetic\_gene\_cluster | 25 | 39.921976593 | 131 | 8.4e-30 |
| CAI47638.1 | putative\_ABC-type\_aminoglycoside\_exporter | BGC0000692 | Apramycin\_biosynthetic\_gene\_cluster | 25 | 33.6150845254 | 131 | 1.1e-29 |
| CAF32376.1 | putative\_ABC\_transporter,\_ATP-binding\_protein | BGC0000712 | Paromomycin\_biosynthetic\_gene\_cluster | 26 | 35.1755526658 | 129 | 2.5e-29 |
| AHZ61877.1 | Lom43 | BGC0000240 | Lomaiviticin\_biosynthetic\_gene\_cluster | 25 | 33.4850455137 | 129 | 4.2e-29 |
| AET14838.1 | multidrug\_resistance\_protein | BGC0001274\_c2 | Non-acetylated\_open-chain\_sophorolipid\_/\_diacetylated\_lactonic | 26 | 36.0858257477 | 129 | 4.2e-29 |
| AAC44070.1 | unknown | BGC0000797 | Sphingan\_polysaccharide\_biosynthetic\_gene\_cluster | 26 | 39.7919375813 | 128 | 5.5e-29 |
| ABP54659.1 | ABC\_transporter\_related | BGC0000241 | Lomaiviticin\_biosynthetic\_gene\_cluster | 26 | 32.3146944083 | 128 | 5.5e-29 |
| WP\_043270371.1 | ABC\_transporter\_ATPase | BGC0001188 | Legonaridin\_biosynthetic\_gene\_cluster | 26 | 33.355006502 | 128 | 5.5e-29 |
| BAB04170.1 | lantibiotic\_mersacidin\_transporter\_system | BGC0000517 | Haloduracin\_alpha\_/\_haloduracin\_beta\_biosynthetic\_gene\_cluster | 23 | 33.6801040312 | 127 | 1.2e-28 |
| AAS92552.1 | SirA | BGC0001044 | Sirodesmin\_biosynthetic\_gene\_cluster | 26 | 35.0455136541 | 126 | 3.5e-28 |
| WP\_010071619.1 | ABC\_transporter\_ATP-binding\_protein | BGC0000578 | SRO15-2005\_biosynthetic\_gene\_cluster | 26 | 32.44473342 | 126 | 3.5e-28 |
| EYT83450.1 | ABC\_transporter | BGC0001213 | Nataxazole\_biosynthetic\_gene\_cluster | 29 | 22.4317295189 | 125 | 4.6e-28 |
| EEM14530.1 | hypothetical\_protein | BGC0001210 | Pseudomycoicidin\_biosynthetic\_gene\_cluster | 26 | 29.7139141743 | 124 | 7.9e-28 |
| BAL72550.1 | lariatin\_biosynthetic\_protein | BGC0000575 | Lariatin\_biosynthetic\_gene\_cluster | 24 | 39.5318595579 | 124 | 1e-27 |
| WP\_006126653.1 | ABC\_transporter | BGC0000554 | SRO15-3108\_biosynthetic\_gene\_cluster | 25 | 36.9310793238 | 124 | 1.3e-27 |
| AHF21240.1 | CrnT | BGC0000500 | Carnolysin\_A1\_/\_carnolysin\_A2\_biosynthetic\_gene\_cluster | 21 | 35.6306892068 | 123 | 2.3e-27 |
| ACA97579.1 | PmxD | BGC0000408 | Polymyxin\_biosynthetic\_gene\_cluster | 24 | 37.9063719116 | 123 | 3e-27 |
| AEZ51519.1 | PmxD | BGC0001153 | Polymyxin\_biosynthetic\_gene\_cluster | 24 | 37.9063719116 | 122 | 3.9e-27 |
| AGM16416.1 | paenibacterin\_ABC-transporter\_E | BGC0000400 | Paenibacterin\_biosynthetic\_gene\_cluster | 25 | 37.0611183355 | 122 | 5.1e-27 |
| AAK73499.1 | AmphH | BGC0000015 | Amphotericin\_biosynthetic\_gene\_cluster | 23 | 30.5591677503 | 121 | 6.7e-27 |
| AFD30962.1 | CrmT3 | BGC0000966 | Caerulomycin\_A\_biosynthetic\_gene\_cluster | 27 | 31.7945383615 | 120 | 1.5e-26 |
| CAP20371.1 | ABC\_transporter\_ATP-binding\_membrane\_protein | BGC0000347 | Equibactin\_biosynthetic\_gene\_cluster | 23 | 34.0052015605 | 120 | 1.9e-26 |
| AJM89737.1 | PmxD | BGC0001192 | Colistin\_biosynthetic\_gene\_cluster | 24 | 37.6462938882 | 119 | 2.5e-26 |
| CAD15511.1 | probable\_composite\_atp-binding\_transmembrane\_abc\_transporter\_protein | BGC0001014 | Micacocidin\_biosynthetic\_gene\_cluster | 27 | 32.5747724317 | 119 | 2.5e-26 |
| CAG38699.1 | putative\_ABC-transporter,\_ATP-binding\_protein | BGC0000708 | Lividomycin\_biosynthetic\_gene\_cluster | 24 | 31.7295188557 | 119 | 4.3e-26 |
| CAC17507.1 | putative\_ABC\_transporter\_ATP-binding\_protein | BGC0000324 | Coelibactin\_biosynthetic\_gene\_cluster | 25 | 36.8660598179 | 118 | 5.7e-26 |
| WP\_009985592.1 | peptidase\_C39 | BGC0001311 | Flavecin\_biosynthetic\_gene\_cluster | 24 | 35.5006501951 | 118 | 5.7e-26 |
| BAH43767.1 | ABC\_transporter\_ATP\_binding/permease\_protein | BGC0000452 | Tyrocidine\_biosynthetic\_gene\_cluster | 24 | 36.0858257477 | 117 | 1.6e-25 |
| AAK27372.1 | ABC\_transporter | BGC0000623 | Plantaricin\_W\_alpha\_/\_plantaricin\_W\_beta\_biosynthetic\_gene\_cluster | 24 | 34.3953185956 | 115 | 6.3e-25 |
| XP\_011392729.1 | ABC\_transporter | BGC0001281 | Ustilagic\_acid\_biosynthetic\_gene\_cluster | 24 | 35.6957087126 | 114 | 1.1e-24 |
| AHW81482.1 | hypothetical\_protein | BGC0000934 | Endophenazines\_biosynthetic\_gene\_cluster | 28 | 21.8465539662 | 111 | 6.9e-24 |
| AFJ14797.1 | PlpH | BGC0000403 | Pelgipeptin\_biosynthetic\_gene\_cluster | 25 | 31.2743823147 | 109 | 3.4e-23 |
| AFN69431.1 | ElxT | BGC0000509 | Epilancin\_15X\_biosynthetic\_gene\_cluster | 22 | 32.1846553966 | 109 | 3.4e-23 |
| EFI65097.1 | lantibiotic\_transport\_protein | BGC0001407 | Bicereucin\_biosynthetic\_gene\_cluster | 24 | 35.5006501951 | 109 | 4.5e-23 |
| ABQ84449.1 | salivaricin\_ABC-transporter | BGC0000624 | Salivaricin\_CRL1328\_alpha\_peptide\_/\_salivaricin\_CRL1328\_beta\_peptide | 24 | 33.0949284785 | 108 | 7.7e-23 |
| CCC55904.1 | putative\_ABC\_transporter,\_ATPase\_and\_permease\_component | BGC0000973 | Collismycin\_A\_biosynthetic\_gene\_cluster | 24 | 33.1599479844 | 106 | 2.2e-22 |
| AEH59056.1 | YvcC/ABC\_transporter | BGC0000385 | Lysobactin\_biosynthetic\_gene\_cluster | 25 | 35.955786736 | 106 | 2.9e-22 |
| ALE27500.1 | ABC\_transporter | BGC0001292 | A33853\_biosynthetic\_gene\_cluster | 27 | 35.5006501951 | 104 | 8.5e-22 |
| AFY23032.1 | TamA | BGC0000274 | Tetarimycin\_biosynthetic\_gene\_cluster | 25 | 25.0325097529 | 104 | 1.4e-21 |
| AAX31526.1 | putative\_ABC\_transporter | BGC0000336 | Daptomycin\_biosynthetic\_gene\_cluster | 24 | 34.5903771131 | 103 | 1.9e-21 |
| AAG32538.1 | SalT | BGC0000548 | Salivaricin\_A\_biosynthetic\_gene\_cluster | 23 | 34.7204161248 | 103 | 1.9e-21 |
| BAB78441.1 |  | BGC0000555 | Staphylococcin\_C55\_alpha\_/\_staphylococcin\_C55\_beta\_biosynthetic\_gene | 23 | 37.5162548765 | 102 | 3.2e-21 |
| AGL93176.1 | ABC\_transporter\_ATP-binding\_protein | BGC0000560 | Subtilomycin\_biosynthetic\_gene\_cluster | 25 | 36.0858257477 | 102 | 4.2e-21 |
| AFD30963.1 | CrmT2 | BGC0000966 | Caerulomycin\_A\_biosynthetic\_gene\_cluster | 27 | 25.4876462939 | 101 | 9.4e-21 |
| CAP20370.1 | ABC\_transporter,\_ATP-binding\_membrane\_protein | BGC0000347 | Equibactin\_biosynthetic\_gene\_cluster | 25 | 24.8374512354 | 99 | 3.6e-20 |
| CAA90021.1 | PepT | BGC0000543 | Pep5\_biosynthetic\_gene\_cluster | 21 | 31.7295188557 | 97 | 1e-19 |
| BAU50927.1 | ABC\_transporter | BGC0001379 | BD-12\_biosynthetic\_gene\_cluster | 24 | 33.6150845254 | 97 | 1.8e-19 |
| ACP43452.1 | McnB | BGC0000590 | Microcin\_N\_biosynthetic\_gene\_cluster | 24 | 31.4044213264 | 95 | 5.1e-19 |
| EYT83451.1 | ABC\_transporter\_ATP-binding\_protein | BGC0001213 | Nataxazole\_biosynthetic\_gene\_cluster | 24 | 32.9648894668 | 92 | 3.3e-18 |
| CAC17506.1 | putative\_ABC\_transporter\_ATP-binding\_protein | BGC0000324 | Coelibactin\_biosynthetic\_gene\_cluster | 25 | 33.2899869961 | 91 | 1.3e-17 |
| ACU73107.1 | ABC\_transporter\_related | BGC0000501 | Catenulipeptin\_biosynthetic\_gene\_cluster | 29 | 21.6514954486 | 90 | 1.7e-17 |
| AAX31527.1 | putative\_ABC\_transporter | BGC0000336 | Daptomycin\_biosynthetic\_gene\_cluster | 27 | 22.5617685306 | 81 | 1e-14 |
| ACU62760.1 | ABC\_transporter\_related | BGC0001392 | Pinensins\_biosynthetic\_gene\_cluster | 23 | 31.9895968791 | 79 | 5e-14 |
| AJF34465.1 | ABC\_transporter | BGC0001207 | Teixobactin\_biosynthetic\_gene\_cluster | 23 | 36.1508452536 | 76 | 4.2e-13 |
| AAC63532.1 | sublancin\_transporter\_protein | BGC0000558 | Sublancin\_168\_biosynthetic\_gene\_cluster | 22 | 35.6957087126 | 74 | 9.4e-13 |
| BAU50928.1 | ABC\_transporter | BGC0001379 | BD-12\_biosynthetic\_gene\_cluster | 24 | 30.2340702211 | 74 | 9.4e-13 |
| CAG34716.1 | putative\_ABC\_transporter | BGC0000713 | Ribostamycin\_biosynthetic\_gene\_cluster | 25 | 30.7542262679 | 72 | 3.6e-12 |
| BAG19224.1 | membrane\_translocator | BGC0000496 | AmfS\_biosynthetic\_gene\_cluster | 22 | 35.6306892068 | 64 | 1.3e-09 |
| AAO23332.1 | NcpC | BGC0000397 | Nostocyclopeptide\_biosynthetic\_gene\_cluster | 22 | 31.5344603381 | 57 | 1.2e-07 |
| WP\_020635024.1 | multidrug\_ABC\_transporter\_ATPase | BGC0001211 | Albachelin\_biosynthetic\_gene\_cluster | 26 | 20.611183355 | 57 | 1.5e-07 |
| CAM02316.1 | ABC\_transporter\_protein,\_ATP-binding\_component | BGC0000349 | Erythrochelin\_biosynthetic\_gene\_cluster | 25 | 20.0910273082 | 57 | 1.5e-07 |
